# Diagnostic Capability of Next-Generation Sequencing Fusion Analysis in Identifying a Rare CASE of *TRAF1-ALK-*Associated Anaplastic Large Cell Lymphoma

**DOI:** 10.3389/fonc.2020.00730

**Published:** 2020-05-08

**Authors:** Indu Agarwal, Linda Sabatini, Mir B. Alikhan

**Affiliations:** Department of Pathology and Laboratory Medicine, NorthShore University HealthSystem, Evanston, IL, United States

**Keywords:** anaplastic T-cell lymphoma, ALK (anaplastic lymphoma kinase), next- generation sequencing, RNA based sequencing, lymphoma - diagnosis

## Abstract

**Background:** Anaplastic lymphoma kinase (ALK)-positive anaplastic large cell lymphoma (ALCL) is a rare T-cell neoplasm, accounting for approximately 3% of adult non-Hodgkin lymphomas. Although NPM1 is the most common fusion partner with ALK, many others have been described, necessitating break-apart FISH studies for confirmation of the diagnosis. TNF receptor-associated factor 1 (TRAF1) is a rare ALK partner that is thought to confer a worse prognosis in patients. We describe the utility of next-generation sequencing (NGS) RNA analysis in detection of this uncommon ALK partner.

**Case Description:** A 42-year-old male with cervical lymphadenopathy presented for excisional biopsy. Following a tissue diagnosis of ALCL, ALK+, RNA from the biopsy was extracted from Formalin-fixed paraffin-embedded (FFPE) tissue and prepared for Anchored Multiplex PCR using the Archer® FusionPlex® v2 assay, which employs unidirectional gene-specific primers using NGS to detect novel or unknown gene partners.

**Results:** Histologic evaluation of the excised lymph node showed atypical cells, including “horseshoe/kidney”-shaped nuclei. Neoplastic cells were immunoreactive against CD30, ALK (diffuse, cytoplasmic), CD2, CD4, granzyme B, and TIA-1. A diagnosis of ALCL, ALK+ was made. The pattern of ALK immunostaining suggested a non-NPM1-associated ALK translocation pattern, prompting further investigation. NGS fusion analysis showed a translocation involving exon 7 of TRAF1 and exon 20 of ALK.

**Conclusion:** ALK positivity suggests an overall favorable prognosis of ALCL as compared to ALK-negative cases. However, in the rare published cases of TRAF1-ALK, an aggressive clinical course has been observed, which may reflect the aggressive propensity of this particular fusion, as these cases appear to be refractory to standard chemotherapy and also to the first generation ALK inhibitors. This study highlights the advantage of using NGS in RNA-based fusion assays to detect rare translocations, which can be of some clinical importance in detecting rare but aggressive fusion partners of ALK. As these technologies become more available, there is potential to identify such changes and effectively stratify the prognosis of ALCL patients.

## Background

In 1995, Stein and colleagues described a set of lymphomas characterized by bizarre morphologic features with large cells and positivity for the Ki-1 (CD30) antigen (1). It was later discovered that a subset of cases harbored the *t*(2,5)(p23;q35) translocation, juxtaposing the *NPM1* gene to the *ALK* gene (2). An antibody against this translocation was subsequently developed and found to be immunoreactive against the variant translocations involving ALK (3). The antibody is also immunoreactive against the chimeric ALK protein harboring other partners (4).

Although the majority of ALK+ ALCL exhibit the NPM1-ALK reciprocal translocation, many variant translocations have been identified. Morphologically and immunohistochemically, there is no significant difference between the NPM1-associated ALK+ ALCL and those with the variant translocations (5). With respect to clinical outcome, most agree that there is no significant difference in survival between the different translocations (6). However, recent reports have characterized an ALK+ ALCL with the tumor necrosis factor-1 associated factor (*TRAF1*) gene translocation. Although only a few cases have been reported, some have exhibited more aggressive clinical features. We highlight in this report another TRAF1-ALK-associated ALK+ ALCL and describe the clinical course of this case. This case also highlights the utility of RNA-based next-generation sequencing (NGS) analysis to detect novel and rare translocations involving genes such as *ALK*.

## Case Presentation

A 42-year-old Caucasian male presented history of progressive fatigue, chills, night sweats, and unwanted weight loss. He was found to have left cervical lymphadenopathy. Magnetic resonance imaging (MRI) revealed widespread adenopathy, innumerable small sclerotic lesions throughout the bones and one small lucent lesion in the right side of the pelvis. He underwent excisional biopsy of the cervical lymph node.

**Figure 1:**
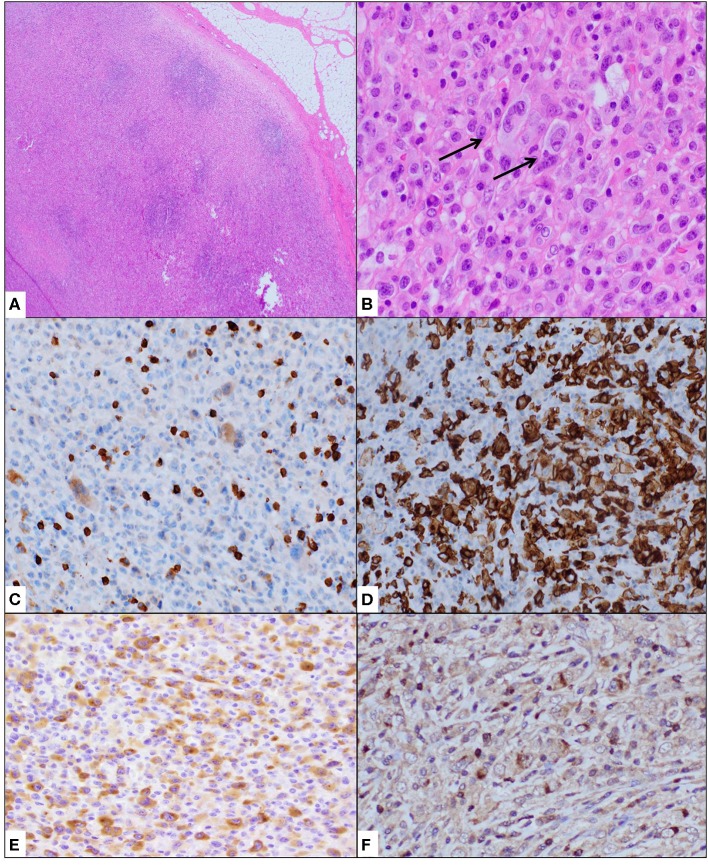
Cervical lymph node biopsy showed effacement of architecture **(A)** with atypical cells, often containing “hallmark” cells with kidney-shaped nuclei **(B)**. Immunohistochemical stains showed these neoplastic cells to be weak or negative for CD3 **(C)** and strongly positive for CD30 **(D)**. ALK1 immunostain showed granular cytoplasmic positivity **(E)** while TIA1 was variably positive **(F)**. Staining for histiocyte CD68 showed increased histiocytes.

Histologic evaluation of the excised lymph node showed effaced architecture with many atypical cells, including those with “horseshoe/kidney”-shaped nuclei. The neoplastic cells were immuno-reactive against CD30, ALK (diffuse, cytoplasmic), CD2, CD4, granzyme B, and TIA-1 (Figure 1). Many background CD68+ histiocytes were noted. A diagnosis of ALCL, ALK+ was made. The pattern of ALK immunostaining suggested a non-NPM1-associated ALK translocation pattern, prompting further investigation. The increased histiocytes were suggestive of a histiocyte-rich variant.

Next-generation sequencing (NGS) fusion analysis utilizing RNA from the sample was performed as described previously (7). Briefly, the tissue was prepared from 10-μM sections of formalin-fixed, paraffin-embedded tissue placed on charged slides. Following deparaffinization, PinPoint RNA Isolation System II (Zymo Research Corp.) was applied to a 1 mm^2^ area of the slide. After drying, this area was removed from the slide and transferred to a sterile tube. The product was treated with proteinase K, ethanol, and RNA extraction buffer. The RNA concentration of the final product was determined by the Qubit fluorometric quantification system (Life Technologies). The target-enriched library was prepared with the Archer FusionPlex Lung/Thyroid Panel (ArcherDX, Boulder, CO) as per manufacturer's instructions (8). This kit contains targets for the ALK gene. Using the anchored multiplex PCR (AMP) design, gene rearrangements, including novel variants, can be detected (9). Sequencing was performed using the IonTorrent S5 system (ThermoFisher, Waltham, MA).

**Figure 2:**
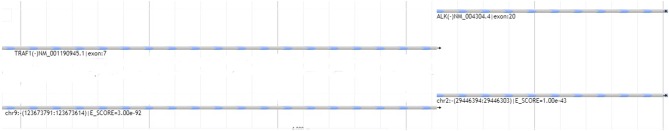
Graphical representation of next-generation sequencing translocation analysis. Reads aligned with exon 20 of *ALK1* were found to be adjacent with reads aligned with exon 7 of the *TRAF1* gene. E-score represents a confidence score whereby lower values reflect higher confidence of sequence alignment in the reads.

**Figure 3:**
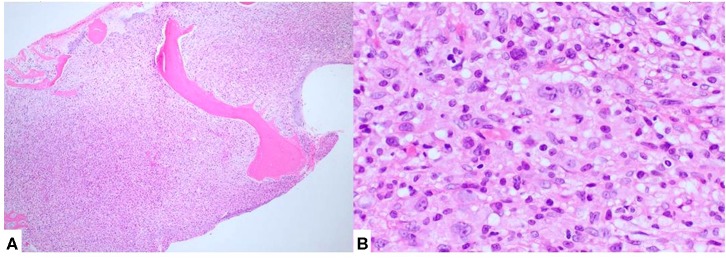
Bone marrow biopsy showed diffuse involvement by lymphoma **(A)**. Atypical cells, morphologically similar to those seen in the lymph node biopsy, were also present **(B)**.

Following confirmation of the diagnosis with NGS translocation analysis (Figure 2), a bone marrow biopsy was performed and showed extensive disease (Figure 3). The patient received 6 cycles of CHOEP chemotherapy, consisting of the following chemotherapy drug(s): Cyclophosphamide, Doxorubicin, Vincristine and Etoposide. This was followed by a repeat bone marrow biopsy at 4 months, which showed a cellular bone marrow with trilineage hematopoiesis without morphologic evidence of residual disease.

To detect molecular residual disease, *TRG* gene rearrangement studies were performed on the bone marrow from the aspirate sample. DNA extraction was performed using Qiagen PureGene kits as described previously (10) (Qiagen, Germantown, MD). *TRG* gene rearrangement analysis was performed by next-generation sequencing using the Lymphotrack NGS assay (Invivoscribe, San Diego, CA) as previously described (11) following the manufacturer's protocol. Briefly, PCR amplification was performed using master mixes with primers derived from barcoded sequence adaptors. The library sequencing was performed using the IonTorrent platform. Results were analyzed using Lymphotrack S5-PGM Software version 2.4.5 (Invivoscribe, Inc.). Determination of clonality in these cases was based on current expert opinion outlined previously (12). Briefly, the total number of sequence reads should exceed 100,000, the dominant sequences should be >2.5% of all reads, and >10-times the polyclonal background.

The initial biopsy showed two dominant clones at 22.5% and 8.6% in an otherwise polyclonal background. The subsequent biopsy showed a mostly polyclonal pattern of rearrangements. Further analysis identified the identical sequences of the dominant clones of the original biopsies present in the re-biopsy at 0.011 and 0.003%, respectively.

The patient was later aggressively treated, starting on high dose BEAM chemotherapy which involved carmustine, etoposide, cytarabine, and melphalan with autologous stem cell reinfusion. The patient is overall doing well 9 months post-transplant.

## Discussion

In 1994, Bullrich and colleagues found that the *NPM1* gene was recurrently translocated with the *ALK* gene in Ki-1 (CD30) positive lymphomas (13). The rearrangement results in an NPM1-ALK fusion protein that leads to constitutive activation of the ALK kinase. The consequence of this is constitutive activation of the ALK kinase and deregulation of multiple signaling pathways downstream of ALK, culminating in increased cellular proliferation and survival (14).

The translocation in question, *t*(2,5)(p23;q35), juxtaposes the chromosomal region 5q35, which includes the *NPM1* gene, with 2p23 which includes the gene encoding for ALK, a receptor tyrosine kinase. This leads to increased expression of ALK. The translocation results in a chimeric gene that encodes an 80-kD fusion protein. This protein combines the N-terminal portion of NPM1 to the entire cytoplasmic domain of the receptor tyrosine kinase ALK (10). Antibodies against ALK used as immunohistochemical stains are reactive against the NPM1-ALK fusion protein as well as the full-length ALK protein, and have been used to detect lymphomas with ALK-associated translocations (15).

In ALK-positive lymphomas with *NPM1-ALK* translocation, strong immunohistochemical staining for ALK protein is not only seen in the cytoplasm of the lymphoma cells, but also within their nuclei and nucleoli. This is likely due to the association with NPM1, which functions to shuttle proteins to the nucleolus. Specifically, wild-type NPM provides nuclear localization signals, whereby the NPM-ALK protein can enter the nucleus (2, 16). In the *t*(2,5)(p23;q35) translocation, the particular cytoplasmic, nuclear, and nucleolar staining can be explained by the formation of dimers between wild-type NPM and the fusion NPM-ALK protein. When the NPM1-ALK fusion protein forms homodimers, the dimerization process mimics ligand binding. This leads to autophosphorylation of the tyrosine kinase domain of ALK and constitutive activation, resulting in the oncogenic properties seen in anaplastic lymphomas (10, 17). After its dimerization, the NPM1-ALK fusion protein activates various signaling pathways, including phosphatidylinositol-3 kinase(PI3K)/AKT/mTOR, phospholipase C (PLCγ), JAK/signal transducer and activator of transcription 3 (STAT3), and RAS/ERK (18–20). The activation of these multiple pathways by the NPM1-ALK fusion protein leads to cell-cycle progression, proliferation, cell survival and anti-apoptotic functions (21).

However, not all ALK-positive lymphomas of carry the *NPM1-ALK* fusion gene and other fusions have since been described. In these variant translocations, often only cytoplasmic ALK staining is observed and nuclear staining is negative. Besides the *t*(2,5) translocation, at least 11 variant translocations involving the *ALK* gene at p23 have been recognized. In all these translocations, the *ALK* gene is placed under the control of the promoter of genes that are constitutively expressed in lymphoid cells leading to elevated *ALK* gene expression. The most common variant translocation is *t*(1,2)(q25;p23), in which the *TPM3* gene, located on chromosome 1, is fused to the *ALK* catalytic domain (4). However, in cases associated with the *TPM3-ALK* translocation, which express the TPM3-ALK fusion protein, measuring 104kDa, ALK staining is often restricted to the cytoplasm of the lymphoma cells. Additionally, most cases show stronger immunostaining of the cell membrane. The *t*(1,2) translocation is found in about 15–20% of ALK+ anaplastic lymphoma cases.

A less common translocation, the *t*(2,3)(p23;q11), involves the *TFG* gene. It produces two different fusion transcripts, of 85 and 97 kDa. These are referred to as TFG-ALK_short_ and TFG-ALK_long_ (22). The inv (2)(p23q35) translocation involves the *ATIC* gene (formerly known as *pur-H*), which codes for a protein involved in purine biosynthesis (26). In both TFG-ALK–positive and ATIC-ALK–positive ALCL cases, ALK staining is limited to the cytoplasm in a diffuse pattern, without nuclear staining. In rare cases of ALCL showing a unique granular ALK cytoplasmic staining pattern, the fusion gene partner is often implicated to be the *CLTC* gene the codes for clathrin heavy chain 1, a protein involved in intracellular trafficking of receptors (27).

Falini et al. described the immunohistologic and clinicopathologic features of 15 ALK-positive lymphoma cases that express ALK fusion protein(s) other than NPM1-ALK. They found that although there are some morphologic differences between non-NPM1-ALK fusion cases from the classical ALK+ ALCL with NPM1-ALK1 fusion, the clinical features are largely equivalent. These cases mostly occurred in children or young adults who presented with advanced disease associated with extranodal involvement and B symptoms (especially fever). Additionally, the overall prognosis of the variant ALK-positive lymphomas was similar to the classical NPM1-ALK cases with regard to relatively superior survival rates. The variant fusion cases were also significantly different in terms of prognosis from ALK-negative ALCLs (17). Other studies have also concluded that no significant differences have been found between NPM1-ALK-positive lymphomas and those showing variant fusions involving *ALK* and partners other than *NPM1* (6) (Table 1).

**Table 1:** Clinical summary and pathology of previously published and current case with ALK-TRAF fusion.

**References**	**Age at presentation**	**Initial presentation**	**Pathologic features and diagnosis**	**Duration of follow up and outcome**
Feldman et al. (23)	41/M	Lymphadenopathy and rash	**Morphology:** Effaced nodal architecture with “hallmark” cells in a background of small lymphocytes, histiocytes, and plasma cells **IHC**: Positive stains: CD30, ALK (cytoplasmic), TIA1, granzyme B, and clusterin Negative stains: CD3, CD4, CD5, CD7, and CD8. **Molecular diagnosis:** Deep RNA sequencing identified the fusion of Exon 6 of TRAF1 to Exon 20 of ALK. The TRAF1-ALK fusion transcript was confirmed at the mRNA level by Sanger sequencing The fusion protein was visualized by Western blot	336 months, patient alive
Takeoka et al. (24)	51/M	Left axillary tumor with infiltration of overlying skin	**Morphology:** Diffuse infiltration in the dermis by large lymphoma cells **IHC**: Positive stains: CD30, EMA, CD4, and ALK (cytoplasmic) Negative stains: CD3, CD8, CD10, CD20, and EBER **Molecular diagnosis:** G-banding and FISH using an *ALK* break-apart probe revealed the rearrangement of *ALK* and suggested variant translocation Nucleotide sequencing of the PCR products and a database search showed the sequences of *TRAF1* following those of *ALK*. Subsequently *TRAF1*-*ALK* fusions confirmed by reverse transcriptase PCR and nucleotide sequencing	15 years (~180 months), patient died of therapy-related acute myeloid leukemia
Lawrence et al. (25)	32/M	Cough, fever, dyspnea, chest pain, weight loss, and extensive lymphadenopathy at cervical, supraclavicular and mediastinal sites	**Morphology:** Complete effacement of the nodal architecture by diffuse infiltration of large and giant pleomorphic cells characterized by conspicuous nucleoli and copious cytoplasm **IHC**: Positive stains: CD30, CD45, CD43, and ALK (cytoplasmic) Negative stains: CD3, CD4, CD5, and CD8 **Molecular diagnosis:**Non-allele-specific ALK RT-qPCR revealed ALK overexpression and 5′ RACE PCR showed the TRAF1-ALK fusion	15 months, patient died
Agarwal, this study*	42/M	Progressive fatigue, chills, night sweats, and weight loss, and cervical lymphadenopathy Imaging showed widespread adenopathy and multiple small bone lesions	**Morphology:** Effaced nodal architecture with many atypical cells, including those with “horseshoe/kidney”-shaped nuclei **IHC**: Positive stains: CD30, ALK (diffuse, cytoplasmic), CD2, CD4, CD45, granzyme B, and TIA-1 Negative stains: CD3, CD20, PAX5, CD8, and CD5 **Molecular diagnosis:** NGS fusion analysis showed translocation involving exon 7 of TRAF1 and exon 20 of ALK	18 months, patient is overall doing well 9 months post-transplant

* *current case*.

*TIA-1, T-Cell Intracellular Antigen 1; CHOP, Cyclophosphamide, doxorubicin, vincristine, and prednisone; CHOEP, Cyclophosphamide, doxorubicin, vincristine, prednisone, and etoposide; ProMACE-CytoBOM, Prednisone, doxorubicin, cyclophosphamide, etoposide, arabinoside cytosine, bleomycin, vincristine, and methotrexate; RACE, Rapid amplification of cDNA ends; PCR, Polymerase chain reaction; DeVIC, Dexamethasone, etoposide, ifosfamide and carboplatin; DHAP, Dexamethasone, cytarabine, and cisplatin; BEAM, Bis-chloroethylnitrosourea, etoposide, cytarabine, and melphalan; GDP, Gemcitabine, dexamethasone, and cisplatin*.

Another non–NPM fusion partner which has been described recently is the tumor necrosis factor receptor (TNFR)-1 associated factor (*TRAF1*) gene. Feldman et al. described a case of a 41-year-old male who suffered from an almost 30-year history of relapses of his lymphoma. He achieved remission only after high-dose chemotherapy and autologous stem cell transplantation (23). Another case was described by Lawrence et al. of a 32-year-old Caucasian male ALCL patient whose lymphoma was refractory to standard chemotherapy and autologous stem cell transplantation. This patient also showed poor response to ALK inhibitor targeted therapy (25). A third case described by Takeoka et al. was of a 51-year-old man with multiple skin tumors who also had a poor response and eventually died (24).

We describe a fourth case of *TRAF1-ALK* translocation. In our case, the patient underwent intensive chemotherapy and early bone marrow transplantation due to aggressive features of his disease. This highlights the importance of elucidating all ALK partner, as some of these cases can have worse prognosis. Despite their potential clinical and prognostic value, there is a paucity of information in the literature about *TRAF1-ALK*.

The TRAF family of proteins functions as adaptor molecules that interact with tumor necrosis factor (TNF) receptors. Once they link with TNFRs, they lead to activation of downstream kinase pathways. Binding of TNF to its receptor leads to recruitment of various TRAF proteins to result in activation of transcription factors such as nuclear factor κB (NF-κB) and activator protein-1 (AP-1) (28–30). To date, there are six members of the TRAF family, TRAF1-6. Yet another interaction is with CD30; the cytoplasmic tail of this protein interacts with TRAFs 1-3 and TRAF5 to result in activation of NF-κB (31, 32). The downstream result of this pathway activation is transcription of anti-apoptotic genes, leading to increased cell survival, protection from apoptosis, and proliferation (31).

In this regard, Abate et al. have studied the properties and functions of the TRAF1-ALK fusion protein. The N-terminal of the TRAF1 region and its interaction with ALK leads to alterations in NF-kB function, contributing to tumorigenesis and maintenance of the neoplastic phenotype (33). The chimeric protein can also associate with TRAF2 in order to alter the activation of NF-kB through the CD30 protein. This study also presented a gene data set that was able to stratify anaplastic large cell lymphoma based on NF-kB, regardless of ALK status. This intriguing model suggests that certain subgroups of ALCL may have different outcomes clinically. Data from such studies may be utilized in the future to target poor responders in ALK+ ALCL. One such option is the use of NF-kB inhibitors to target lymphoma cells or the host environment. The latter target is also significant as some studies suggest the NF-kB pathway may be relevant in host cells as well. Thus, targeting the tumor microenvironment is also a potential strategy in treating ALCL patients (34).

The popular notion that all ALK+ ALCLs behave in a similar fashion clinically has precluded the practical need for identification of the ALK partner gene. In this regard, most diagnoses of ALK+ ALCL can be confirmed by break-apart FISH analysis, such as in the more common *t*(2,5) translocation involving *NPM1-ALK. One* methodology that has been developed in cases when the identification of the partner gene is desired is rapid amplification of cDNA ends (RACE) (23). This technology utilizes a 50-gene panel to identify most ALK partner genes and has successfully identified some partners that are often cytogenetically cryptic, as in the case of the *ATIC-ALK* translocation (26).

The advent of next-generation sequencing and other deep sequencing platforms have allowed the rapid discovery of unique and novel translocations in cancer. However, this technology has not been utilized widely in the detection of translocations in ALK+ ALCLs. Here, we employed next-generation sequencing in an RNA-based fusion assay to detect this rare translocation partner. The advantage of the Archer assay is the ability to detect novel translocation partners for ALK in the case of ALCLs and lung carcinomas as well as other translocations in many other tumors. As NGS is employed increasingly in clinical and research settings, one may expect further studies investigating the biologic and clinical significance of these genetic lesions. Based on a cost prospective, it may be prohibitive to employ such testing on all ALK+ cases. It may also be possible to design a dual-fusion FISH probe to detect the specific *TRAF1-ALK* fusion. However, the utility of NGS is the discovery of novel fusions which may, in the future, be associated with adverse prognosis.

ALK positivity suggests an overall favorable prognosis of ALCL as compared to ALK-negative cases. However, in the rare published cases of *TRAF1-ALK*, an aggressive clinical course has been observed, which may reflect the aggressive propensity of this particular fusion, as these cases appear to be refractory to standard chemotherapy and also to first generation ALK inhibitors. In our case, aggressive chemotherapy and bone marrow transplantation likely aided in remission of disease. The current modalities to detect *ALK* translocation are IHC and FISH, but neither would identify these rare *ALK* partners. This study highlights the utility of using NGS in RNA-based fusion assays to detect rare translocations, which can be of clinical importance in detecting rare but aggressive fusion partners of *ALK*, e.g., *TRAF*. As these technologies become more available, there is potential to identify such changes and effectively stratify the prognosis of ALCL patients.

## Data Availability Statement

The original contributions presented in the study are publicly available. This data can be found here: the NCBI GeneBank (www.ncbi.nlm.nih.gov/nuccore/MT163661).

## Author Contributions

MA and IA contributed to the design of the study, review of relevant clinical and pathologic data, and development of the manuscript. LS provided technical review of the data and editing of the manuscript.

## Conflict of Interest

The authors declare that the research was conducted in the absence of any commercial or financial relationships that could be construed as a potential conflict of interest.
